# Adverse maternal and perinatal outcomes in adolescent pregnancies: The Global Network’s Maternal Newborn Health Registry study

**DOI:** 10.1186/1742-4755-12-S2-S8

**Published:** 2015-06-08

**Authors:** Fernando Althabe, Janet L Moore, Luz Gibbons, Mabel Berrueta, Shivaprasad S Goudar, Elwyn Chomba, Richard J Derman, Archana Patel, Sarah Saleem, Omrana Pasha, Fabian Esamai, Ana Garces, Edward A Liechty, K Michael Hambidge, Nancy F Krebs, Patricia L Hibberd, Robert L Goldenberg, Marion Koso-Thomas, Waldemar A Carlo, Maria L Cafferata, Pierre Buekens, Elizabeth M McClure

**Affiliations:** 1 Institute for Clinical Effectiveness and Health Policy, Buenos Aires, Argentina; 2 RTI International, Durham, NC, USA; 3 KLE University’s Jawaharlal Nehru Medical College, Belgaum, India; 4 University Teaching Hospital, University of Zambia, Lusaka, Zambia; 5 Christiana Care, Newark, DE, USA; 6 Indira Gandhi Government Medical College and Lata Medical Research Foundation, Nagpur, India; 7 Department of Community Health Sciences, Aga Khan University, Karachi, Pakistan; 8 Moi University School of Medicine, Eldoret, Kenya; 9 Fundación para la Alimentación y Nutrición de Centro América y Panamá (FANCAP), Guatemala City, Guatemala; 10 Indiana University School of Medicine, Indianapolis, IN, USA; 11 University of Colorado School of Medicine, Denver, CO, USA; 12 Massachusetts General Hospital for Children, Boston, MA, USA; 13 Department of Obstetrics/Gynecology, Columbia University, New York, NY, USA; 14 Eunice Kennedy Shriver National Institute of Child Health and Human Development, Bethesda, MD, USA; 15 University of Alabama at Birmingham, Birmingham, AL, USA; 16 UNICEM, Buenos Aires, Argentina; 17 Tulane School of Public Health and Tropical Medicine, New Orleans, LA, USA

**Keywords:** adolescent pregnancy

## Abstract

**Background:**

Adolescent girls between 15 and 19 years give birth to around 16 million babies each year, around 11% of births worldwide. We sought to determine whether adolescent mothers are at higher risk of maternal and perinatal adverse outcomes compared with mothers aged 20–24 years in a prospective, population-based observational study of newborn outcomes in low resource settings.

**Methods:**

We undertook a prospective, population-based multi-country research study of all pregnant women in defined geographic areas across 7 sites in six low-middle income countries (Kenya, Zambia, India, Pakistan, Guatemala and Argentina). The study population for this analysis was restricted to women aged 24 years or less, who gave birth to infants of at least 20 weeks’ gestation and 500g or more. We compared adverse pregnancy maternal and perinatal outcomes among pregnant adolescents 15-19 years, <15 years, and adults 20-24 years.

**Results:**

A total of 269,273 women were enrolled from January 2010 to December 2013. Of all pregnancies 11.9% (32,097/269,273) were in adolescents 15-19 years, while 0.14% (370/269,273) occurred among girls <15 years. Pregnancy among adolescents 15-19 years ranged from 2% in Pakistan to 26% in Argentina, and adolescent pregnancies <15 year were only observed in sub-Saharan Africa and Latin America. Compared to adults, adolescents did not show increased risk of maternal adverse outcomes. Risks of preterm birth and LBW were significantly higher among both early and older adolescents, with the highest risks observed in the <15 years group. Neonatal and perinatal mortality followed a similar trend in sub-Saharan Africa and Latin America, with the highest risk in early adolescents, although the differences in this age group were not significant. However, in South Asia the risks of neonatal and perinatal death were not different among adolescents 15-19 years compared to adults.

**Conclusions:**

This study suggests that pregnancy among adolescents is not associated with worse maternal outcomes, but is associated with worse perinatal outcomes, particularly in younger adolescents. However, this may not be the case in regions like South Asia where there are decreasing rates of adolescent pregnancies, concentrated among older adolescents. The increased risks observed among adolescents seems more likely to be associated with biological immaturity, than with socio-economic factors, inadequate antenatal or delivery care.

**Trial registration number:**

NCT01073475

## Background

Adolescent girls between 15 and 19 years give birth to around 16 million babies each year, around 11% of births worldwide. Ninety-five percent of these births occur in low- and middle-income countries, where complications from pregnancy and childbirth are a leading cause of death among girls of this age [1]. Although fertility rates in adolescents have declined since 1990, progress has slowed in this century, mainly in sub-Saharan Africa and Latin America, where about half and one third of women give birth before the age of 20, respectively [2,3].

Pregnancy in adolescence has been associated with an increased risk of adverse pregnancy outcomes such as preterm birth [4-9], low birth weight (LBW) [4-9], perinatal death [5-8,10], obstructed labor [3,11], and maternal deaths [1,4]. However the evidence is still controversial; the extent to which the observed associations were caused by the biological immaturity of the adolescent mothers, or were confounded by their frequently poor socioeconomic conditions and lack of health care is still a matter of debate [4,5,7,8,12-14].

This conflicting evidence of research on adolescent pregnancy outcomes to date may be explained by the heterogeneity of study settings (hospital-based vs population-based studies), small sample sizes, especially for adolescents <15 years, and different conceptual approaches to adjust for potential confounders [4,5].

Hence, there is a need for high quality, large, population-based studies, especially from low resource settings where most of the adverse outcomes occur. We aim to determine whether adolescent mothers are at higher risk of maternal and perinatal adverse outcomes compared with mothers aged 20–24 years in a prospective, population-based study of newborn outcomes in low resource settings, the Global Network for Women’s and Children’s Health Research Maternal Newborn Health Registry (MNHR) [15].

## Methods

### The Global Network’s Maternal Newborn Health Registry (MNHR)

The MNHR is a prospective, population-based observational study that includes all pregnant women and their outcomes in defined geographic communities (clusters). These clusters with approximately 300 – 500 annual births were established in health districts by 7 research sites in western Kenya (Moi University), Kafue and Chongwe, Zambia (University of Zambia), Thatta, Pakistan (Aga Khan University) Belgaum, India (KLE University), Nagpur, India (Indira Gandhi University), Chimaltenango Guatemala (FANCAP), and Corrientes and Santiago del Estero Argentina (IECS). The MNHR was initiated at each of the study sites between 2009 and 2010 and continues to the present.

Registry administrators (RA’s), paid study staff who were usually community health workers, nurses, or midwives, identified pregnant women and generally consented those who were eligible by 20 weeks gestation. All women who were residents of the defined communities were eligible and contacted. The RA’s then obtained basic health information at enrollment, and conducted a follow-up visit at or following delivery to collect pregnancy outcomes and health care provided during delivery. A second follow up visit at or after 42 days was done to collect data on maternal and infant health status. Information on the study outcomes was based on medical record review, and birth attendant and family interviews.

All study data were collected, reviewed, and edited by staff at each study site. Data were then transmitted to a central data-coordinating center (RTI International, Durham NC) using a secure process, with additional edits performed centrally and addressed at each site.

The MNHR study was reviewed and approved by all sites’ ethics review committees (CEMIC, Buenos Aires, Argentina; Francisco Marroquin University, Guatemala; University of Zambia, Zambia; Moi University, Kenya; Aga Khan University; KLE University’s Jawharal Nehru Medical College, Belgaum; Indira Gandhi Medical College, Nagpur), the institutional review boards at each U.S. partner university and the data coordinating center (RTI). All women provided informed consent for data collection and follow-up visits. A detailed description of the MNHR methods can be found elsewhere [15].

### Study population

The study population for this analysis was restricted to women enrolled in the MNHR, aged 24 years or less, who gave birth to infants of at least 20 weeks’ gestation and weighing 500g or more. The study period included women enrolled with deliveries January 2010 through December, 2013.

### Exposure and hierarchical approach for the selection of confounders

**Figure 1:**
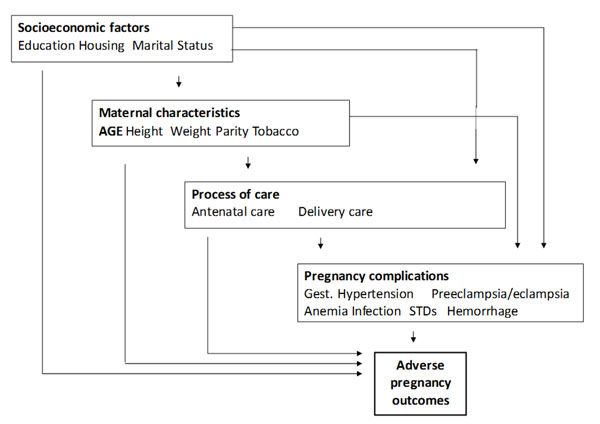
Simplified Conceptual hierarchical framework

The exposure of interest was adolescent maternal age at enrollment categorized into two groups: <15 years (early adolescence), and 15-19 years (older adolescence). The World Health Organization (WHO) defines adolescents as those aged 10 to 19 years [3]. Mothers in the age category 20-24 years were the reference group. As the aim of this study was explanatory, the selection of confounders was based on a conceptual hierarchical framework oriented to distinguish potential confounding factors from mediating factors [16]. Maternal education and parity are distal socioeconomic and reproductive factors of adverse pregnancy outcomes that are also associated with adolescent pregnancy [^3^]. Although data on family income are not collected in the MNHR, the clusters are located in low resource settings in which the vast majority of women are of low socioeconomic condition. Maternal height and pre-pregnancy weight in the Registry had differential missing rates that did not permit these to be included in this analysis. Low pre-pregnancy maternal body mass index (BMI) is associated with preterm birth and LBW, and adolescents tend to have lower BMIs than their adult counterparts [13]. However, low BMI may also be an indicator of biological immaturity in adolescent girls; thus if adolescence was a risk factor of adverse pregnancy outcomes, BMI might be more a mediator in the causal pathway than a confounder, and would not be used for adjustment. Similarly, adolescents may have a different access to, and quality of antenatal and delivery care than the adult mothers. An adverse outcome could be, partially at least, mediated by a lower access to care or lower quality of care. Thus the comparison of the antenatal and delivery care processes between adolescents and adults would be used for the interpretation of the mechanism of action of maternal age on adverse outcomes, rather than as potential confounders. Therefore, to control for confounding, if parity and education were clinically different among the maternal age groups in the univariate analysis we would adjust for these factors in the multivariate analysis. Figure 1 shows a simplified conceptual hierarchical model of the relationships between age and other factors with adverse pregnancy outcomes, adapted from Victora et al [16].

### Maternal and perinatal outcomes

We considered the following maternal outcomes: antepartum and postpartum hemorrhage, obstructed labor, hypertensive disorders, maternal sepsis, and maternal mortality at 42 days postpartum. The perinatal outcomes were: preterm birth (live birth at <37 weeks’ gestation), LBW (live birth weighing <2,500g at birth), stillbirth (fetal deaths occurring >500 g [or >22 weeks gestation]), early neonatal deaths (neonatal deaths 0-6 days after birth), neonatal deaths (neonatal deaths 0-28 days after birth), perinatal deaths (neonatal deaths 0-6 days plus stillbirths).

### Statistical analysis

Descriptive analyses included calculating the frequency and distribution of values. We compared the frequency of maternal characteristics and the process of antenatal and delivery care between the adolescent groups and adults. The interpretation of the differences was done on clinical basis, acknowledging that with these large sample sizes, small but clinically not relevant differences would be statistically significant. To estimate the effect of the adolescent age categories on maternal and perinatal outcomes, generalized linear models were used evaluate the relationship of adolescent age and adverse pregnancy outcomes and to develop point and interval estimates of the relative risk associated with these risk factors; generalized estimating equations were used to account for the correlation of outcomes within cluster in developing appropriate p-values and confidence intervals. All data were analyzed using SAS v.9.3 (Cary, NC).

## Results

**Figure 2:**
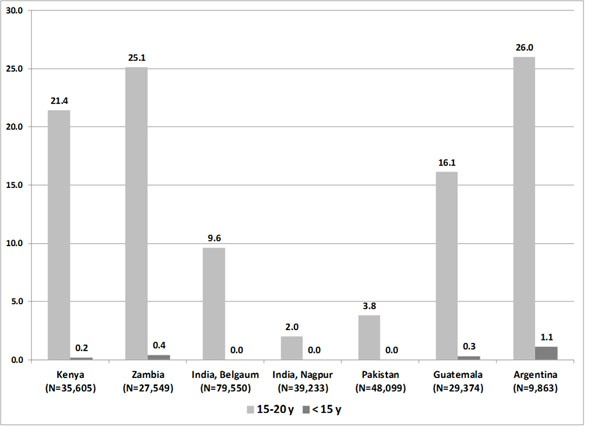
Proportion (%) of births among adolescent mothers 15 – 19 years old and < 15 years old, by GN Site

A total of 269,273 women were enrolled from January 2010 to December 2013. Maternal age was missing for 437 women (0.16%) with no site exceeding 0.4%. The proportion of all pregnancies occurring in adolescents 15-19 years was 11.9% (32,097/269,273), while in girls <15 years it was 0.14% (370/269,273). However, the distribution among sites was very heterogeneous (figure 2). While the proportion of all births in adolescents 15-19 years in the sub-Saharan African (SSA) and Latin American (LA) sites ranged from 16.1% (Guatemala) to 26.0% (Argentina), in the South Asian (SA) sites this proportion ranged from 2.0% (Nagpur, India) to 9.6% (Belgaum, India). The mean age of the adolescents 15-19 years was 18.7 (SD 0.6) years and 17.7 (SD 1.2) in the south Asian sites and sub-Saharan/Latin American sites respectively. Moreover, the proportion of births to adolescents <15 years ranged from 0.2% (Kenya) to 1.1% (Argentina), while there were virtually 0% (10 women) at the three south Asian sites altogether. Due to this heterogeneity, the comparison of the characteristics and outcomes between the adolescents and adults was difficult to interpret when presented altogether. Additionally, the evaluation of adolescents <15 years was not possible at the south Asian sites due to the small sample size. For these reasons only, the results are shown separately in two subgroups: the four SSA and LA sites, and the three SA sites. The comparison of adolescents <15 years was done for the SSA and LA sites only.

Table 1 shows the maternal demographic and obstetric characteristics of the groups of adolescents and adults, stratified by the two subgroups of sites. Compared to women 20-24 year, adolescents 15-19 years showed a slight trend to be less educated, however the differences were not clinically relevant at any site. Adolescents 15-19 years of age were more likely to be nulliparous in both groups of sites. Adolescents <15 years were less educated than older adolescents and adults, but the frequency of women without formal education was <10% in the three age groups.

**Table 1:** Maternal characteristics and pregnancy care

	South Asian Sites (N=166,882)		Sub-Saharan African and Latin American Sites (N=102,391)		
	
	Maternal Age (years)		Maternal Age (years)		
	
	15-19	20-24	< 15	15-19	20-24
Mothers, N	10,233 (6.1)	87,551 (52.5)	360 (0.4)	21,864 (21.4)	33,321 (32.5)

**Maternal characteristics**					

Education, N (%)	10,174	87,147	356	21,804	33,238

No formal education	2,214 (21.8)	17,568 (20.2)	20 (5.6)	1,039 (4.8)	2,412 (7.3)

Primary	3,127 (30.7)	22,248 (25.5)	285 (80.1)	14,275 (65.5)	20,600 (62.0)

Secondary	4,153 (40.8)	36,976 (42.4)	51 (14.3)	6,409 (29.4)	9,556 (28.8)

University+	680 (6.7)	10,355 (11.9)	0 (0.0)	81 (0.4)	670 (2.0)

Parity, N (%)	10,124	87,192	359	21,832	33,288

0	9,491 (93.7)	45,200 (51.8)	347 (96.7)	16,158 (74.0)	8,785 (26.4)

1 or 2	614 (6.1)	40,458 (46.4)	12 (3.3)	5,495 (25.2)	19,843 (59.6)

> 2	19 (0.2)	1,534 (1.8)	0 (0.0)	179 (0.8)	4,660 (14.0)

Last pregnancy resulted in a pregnancy loss, n/N (%)	110/628 (17.5)	2,025/41,962 (4.8)	2/12 (16.7)	672/5,666 (11.9)	1,565/24,485 (6.4)

**Antenatal care (ANC) current pregnancy**					

At least four ANC visits, n/N (%)	2,980/5,016 (59.4)	28,317/45,065 (62.8)	80/181 (44.2)	5,942/11,646 (51.0)	9,708/18,403 (52.8)

Trimester for first ANC visit, N (%)	9.765	84.119	332	20.938	31.912

First	6,213 (63.6)	53,581 (63.7)	73 (22.0)	3,761 (18.0)	5,959 (18.7)

Second	2,653 (27.2)	23,494 (27.9)	183 (55.1)	12,554 (60.0)	18,326 (57.4)

Third	899 (9.2)	7,044 (8.4)	76 (22.9)	4,623 (22.1)	7,627 (23.9)

**Mothers receiving any of the following during this pregnancy**					

Tetanus toxoid vaccine, n/N (%)	9,583/10,228 (93.7)	82,901/87,474 (94.8)	328/359 (91.4)	20,360/21,799 (93.4)	29,379/33,255 (88.3)

Prenatal vitamins/iron, n/N (%)	9,735/10,216 (95.3)	83,660/87,380 (95.7)	316/359 (88.0)	20,531/21,808 (94.1)	31,140/33,274 (93.6)

Syphilis test, n/N (%)	1,539/10,094 (15.2)	21,619/86,658 (24.9)	275/360 (76.4)	16,581/21,793 (76.1)	24,155/33,213 (72.7)

HIV test, n/N (%)	8,269/10,171 (81.3)	75,671/87,141 (86.8)	305/360 (84.7)	18,719/21,794 (85.9)	27,339/33,201 (82.3)

**Delivery care**					

Birth attendant, N (%)	10,229	87,504	360	21,861	33,318

Physician	5,930 (58.0)	49,324 (56.4)	137 (38.1)	4,505 (20.6)	6,490 (19.5)

Nurse/Midwife/HW	3,431 (33.5)	30,153 (34.5)	134 (37.2)	8,397 (38.4)	10,842 (32.5)

TBA	697 (6.8)	6,052 (6.9)	74 (20.6)	7,324 (33.5)	13,082 (39.3)

Family/Other	171 (1.7)	1,975 (2.3)	15 (4.2)	1,635 (7.5)	2,904 (8.7)

Delivery location, N (%)	10,219	87,480	360	21,861	33,318

Hospital	7,043 (68.9)	56,352 (64.4)	191 (53.1)	6,873 (31.4)	9,134 (27.4)

Clinic	2,294 (22.4)	22,744 (26.0)	83 (23.1)	6,206 (28.4)	8,243 (24.7)

Home/Other	882 (8.6)	8,384 (9.6)	86 (23.9)	8,782 (40.2)	15,941 (47.8)

Table 1 also describes the characteristics of the antenatal and delivery care. The frequency of women attending at least four ANC visits did not show clinically relevant differences between adolescents 15-19 years and adults 20-24 years. The gestational age (GA) at first visit was similar in these two groups of women. Adolescents <15 years showed a slightly lower frequency of attendance at four visits, and a similar distribution of GA at first visit, compared to older adolescents and adults. The quality of the ANC was measured through the frequency of preventive interventions and screening procedures. The frequency of tetanus toxoid and preventive iron or vitamins was similar in adolescents 15-19 years and adults in the south Asian sites, and slightly higher in adolescents 15-19 years than in adults in the sub-Saharan/Latin American sites. The proportion of adolescents 15-19 years screened for syphilis and HIV was lower than adults at the SA sites, and slightly higher at the SSA/LA sites. Similarly, early adolescents were slightly more likely to receive screening procedures compared to adults, but these differences were not clinically important.

The birth attendants were similar in adolescents 15-19 years and adults in both groups of sites. However the adolescents were more likely to deliver at hospitals and less likely to deliver at home. This difference was larger in the SSA/LA sites than in SA sites.

**Figure 3:**
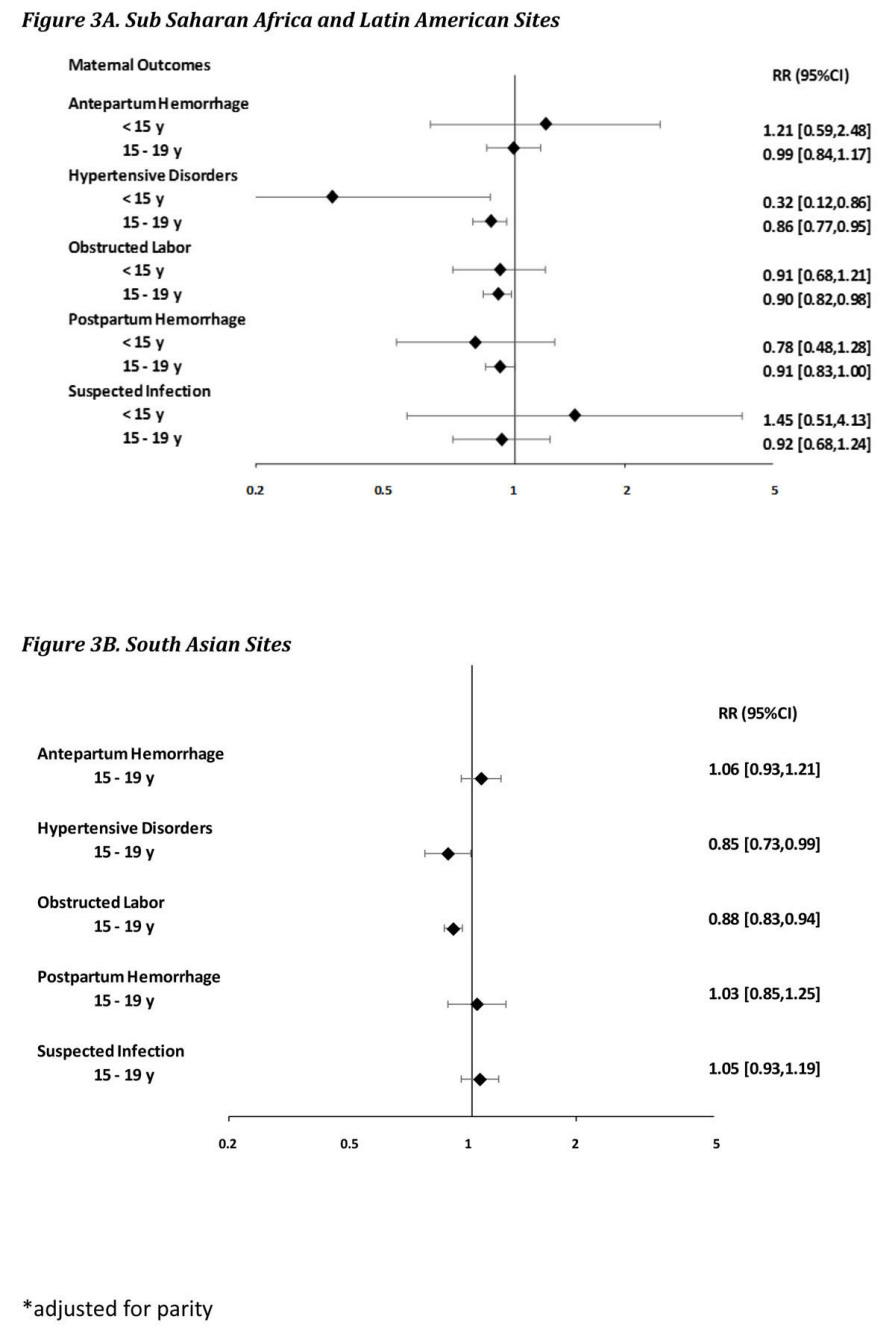
Maternal outcomes Risk Ratios 95% CI’s*

Table 2 describes the maternal and perinatal outcomes rates, by maternal age group and site, and Figure 3 the relative risks and 95% CIs, adjusted for study cluster and parity. The prevalence of antepartum hemorrhage was slightly higher in adolescents 15-19 years than in adults at both SA sites and SSA/LA sites. However, when adjusted by cluster design and parity, there was no statistically significant association in either group [RR 0.99, 95% CI 0.84-1.17 (SA sites); RR 1.06, 95% CI 0.93-1.21 (SSA/LA sites)]. Postpartum hemorrhage showed similar prevalence in older adolescents and adults, in both sites. After adjustment, while there was no association at the SA sites (RR 1.03, 95% CI 0.85-1.05), at the SSA/LA sites, a slightly lower and marginally significant risk in older adolescents was observed (RR 0.91, 95% CI 0.83-1.00). Similar patterns were observed with hypertensive disorders and obstructed labor; while the rates were similar or slightly higher in older adolescents and adults at both groups of sites, after adjustment by cluster and parity, a statistically significant lower risk was observed [hypertensive disorders: RR 0.85, 95% CI 0.73-0.99 (SA sites); RR 0.86, 95% CI 0.77-0.95 (SSA/LA sites); obstructed labor RR 0.88, 95% CI 0.83-0.94 (SA sites); RR 0.90, 95% CI 0.82-0.98 (SSA/LA sites). Finally, maternal sepsis showed no association in older adolescents [RR 1.05, 95% CI 0.93-1.19 (SA sites); RR 0.92, 95% CI 0.68-1.24 (SSA/LA sites)].

**Table 2:** Maternal and perinatal outcomes

	South Asian Sites (N=166,882)		Sub-Saharan African and Latin American Sites (N=102,391)		
	
	Maternal Age (years)		Maternal Age (years)		
	
	15-19	20-24	< 15	15-19	20-24
Deliveries, N	10,233 (6.1)	87,551 (52.5)	360 (0.4)	21,864 (21.4)	33,321 (32.5)

					

**Maternal outcomes**					

Antepartum hemorrhage, N (%)	147/10,188 (1.4)	881/87,395 (1.0)	7/360 (1.9)	385/21,830 (1.8)	491/33,289 (1.5)

Hypertensive disorders, N (%)	271/10,180 (2.7)	2,252/87,340 (2.6)	4/360 (1.1)	462/21,808 (2.1)	635/33,248 (1.9)

Obstructed labor, N (%)	1,426/10,190 (14.0)	10,871/87,355 (12.4)	39/360 (10.8)	2,073/21,819 (9.5)	2,564/33,274 (7.7)

Postpartum hemorrhage, N (%)	68/10,189 (0.7)	493/87,398 (0.6)	7/360 (1.9)	630/21,823 (2.9)	1,025/33,280 (3.1)

Suspected infection (sepsis), n/N (%)	65/10,070 (0.6)	313/86,385 (0.4)	2/359 (0.6)	87/21,706 (0.4)	143/33,105 (0.4)

42-day maternal mortality ratio, n/N (rate/100,000 LB)	19/9,965 (191)	124/85,653 (145)	0/356 (0)	17/21,546 (79)	21/33,028 (64)

**Fetal/Neonatal outcomes**					

Births, N	10,292	88,175	361	21,970	33,558

Preterm birth, n/N (%)	1,211/10,087 (12.0)	9,035/86,945 (10.4)	57/309 (18.4)	2,425/21,073 (11.5)	2,856/32,421 (8.8)

LBW < 2500g, n/N (%)	1,911/10,266 (18.6)	14,106/87,866 (16.1)	50/361 (13.9)	1,900/21,930 (8.7)	2,177/33,508 (6.5)

Stillbirths, n/N (rate/1,000)	323/10,288 (31.4)	2,477/88,130 (28.1)	5/361 (13.9)	422/21,968 (19.2)	527/33,555 (15.7)

Live births, n/N (%)	9,965/10,269 (97.0)	85,653/87,923 (97.4)	356/361 (98.6)	21,546/21,965 (98.1)	33,028/33,547 (98.5)

Neonatal mortality 7 day, n/N (rate/1,000)	281/9,938 (28.3)	1,836/85,411 (21.5)	8/355 (22.5)	325/21,409 (15.2)	383/32,841 (11.7)

Neonatal mortality 28 day, n/N (rate/1,000)	336/9,938 (33.8)	2,260/85,411 (26.5)	9/355 (25.4)	412/21,409 (19.2)	491/32,841 (15.0)

Perinatal mortality 7 day, n/N (rate/1,000)	604/10,261 (58.9)	4,313/87,888 (49.1)	13/360 (36.1)	747/21,831 (34.2)	910/33,368 (27.3)

Similar trends were observed in adolescents <15 years, with wider CIs. Compared to adults 20-24 years and after adjustment for cluster and parity, being in the early adolescent group was not statistically significant in associations for antepartum hemorrhage (RR 1.21, 95% CI 0.59-2.48); post-partum hemorrhage (RR 0.78, 95% CI 0.48-1.28); obstructed labor (RR 0.91, 95% CI 0.68-1.21); and sepsis (RR 1.45, 95% CI 0.51-4.13). Compared to adults, adolescents <15 years showed a statistically significant lower risk of hypertensive disorders (RR 0.32, 95% CI 0.12-0.86).

Preterm birth and LBW rates were higher in adolescents 15-19 years compared to adults, at both SA and SSA/LA sites. After adjustment for cluster (due to the cluster design of the Registry) and parity, older adolescents showed a small but statistically significant increase in the risk of preterm birth [RR 1.15, 95% CI 1.08-1.22 (SA sites); RR 1.23 (95% CI 1.17, 1.30) (SSA/LA sites)], and of LBW deliveries [RR 1.08, 95% CI 1.00-1.15 (SA sites); RR 1.18, 95% CI 1.10-1.27 (SSA/LA sites)].

Although stillbirth rates were slightly higher among adolescents 15-19 years, after adjustment, no difference was observed in SA sites (RR 0.98, 95% CI 0.91-1.06), and a small and not statistically significant increase in risk at SSA/LA sites (RR 1.10, 95% CI 0.95-1.27). Neonatal mortality rates at 28 days showed a somewhat similar pattern. While the rates in adolescents 15-19 years were higher compared to adults at both groups of sites, after adjustment that difference was smaller and not significant in SA sites (RR 1.07, 95% CI 0.97-1.19). Conversely, the relative risk in SSA/LA sites was higher and statistically significant (RR 1.18, 95% CI 1.04-1.33). Neonatal mortality at 7 days followed a very similar same pattern. Finally, after adjustment, perinatal mortality was similar in older adolescents and adults at the SA sites (RR 1.03, 95% CI 0.96-1.10), while significantly higher in older adolescents at the SSA/LA sites (RR 1.03, 95% CI 1.02-1.25).

**Figure 4:**
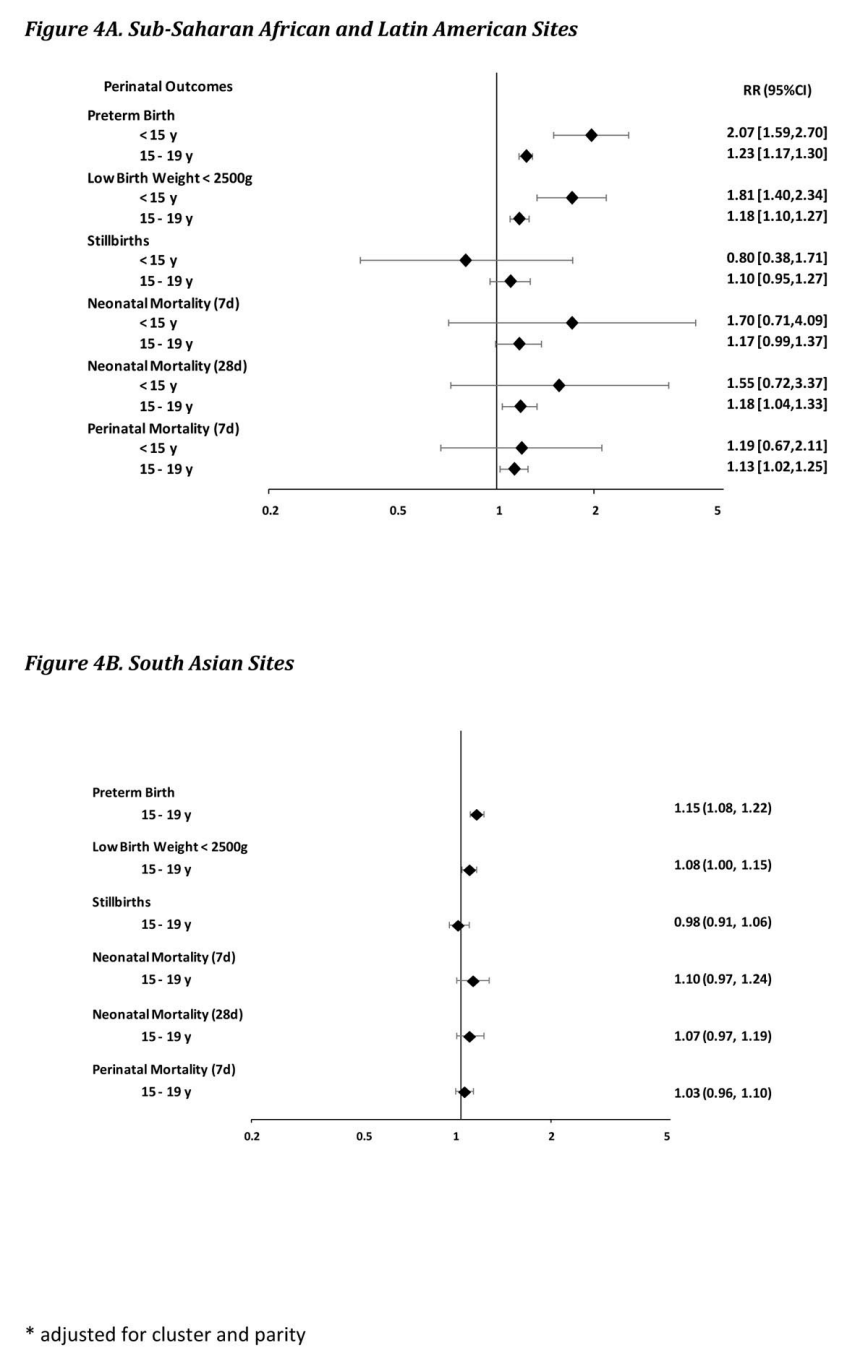
Perinatal outcomes Risk Ratios, 95% CI*

Adolescents <15 years showed even larger relative risks than older adolescents and adults for preterm birth (RR 2.07, 95% CI 1.59-2.70) and LBW (RR 1.81, 95% CI 1.40-2.34). Similar trends were observed in neonatal mortality at 28 days and perinatal mortality, although the differences were not statistically significant. There was no significant association between early adolescence and stillbirths (Table 2, figure 4).

## Discussion

In this large population-based study conducted in six large middle income countries (LMIC), the prevalence of adolescent pregnancy was heterogeneous among regions; while in India and Pakistan the prevalence did not exceed 10%, in sub-Saharan African and Latin American sites it ranged from 16% to 27%. Early adolescent pregnancies were practically nonexistent in the SA region, but were still between 0.2% and 1% in the SSA and LA sites. Adolescents did not show worse maternal outcomes than adults 20-24 years. After controlling for parity and cluster design, we found that there were neither clinical nor statistical significant differences in the risk of antepartum hemorrhage, postpartum hemorrhage, or maternal sepsis among adolescents 15-19 years and <15 years compared to adults 20-24 years. However, the risks of obstructed labor and hypertensive disorders were significantly lower among adolescents 15-19 years compared to adults 20-24 years, although the difference was small. Early adolescents showed similar trends but no significant differences.

Overall, perinatal outcomes showed a more heterogeneous pattern. Risks of preterm birth and LBW were significantly higher among both early and older adolescents, with the highest risks observed in the <15 year old group. Neonatal and perinatal mortality showed to follow a similar trend in SSA and LA, with the highest risk in early adolescents, although the differences in this age group were not significant. However, in south Asia the risks of neonatal and perinatal death were not different among adolescents 15-19 years compared to adults 20-24 years. Finally, stillbirth risks did not differ significantly by adolescent age category.

The study has several strengths. The MNHR is one of the largest prospective population-based studies of maternal and perinatal data in low-resource settings, enrolling more than 250,000 pregnant women in seven sites in six LMICs in four years. The large sample size and a 98% follow-up to obtain pregnancy outcomes, resulted in enough power to detect small differences in perinatal mortality. Additionally, the population-based nature of the registry makes the findings more generalizable than facility-based studies. However, the study has several limitations. The MNHR was not designed to specifically evaluate the association between age and adverse pregnancy, thus not all relevant variables to assess confounding were collected, i.e., marital status and tobacco exposure. However, the lack of these variables has limited impact as adolescent pregnancies out of the wedlock are rare in India and Pakistan [10] and also infrequent in SSA [3]. Additionally, in a previous Global Network study, tobacco exposure during pregnancy has shown to be very low in all sites but Argentina [17]. Another limitation was the quality of the maternal morbidity data, which in these low-resource community settings with a substantial proportion of home births may underestimate the true prevalence, which is reflected by the low rates of hypertensive disorders. Finally, we did not adjust by maternal education in the multivariate model, as education was not clinically relevant, and furthermore, higher education levels were observed among adolescents compared to young adults in the SSA and LA sites. This is consistent with another recent multi-country study [6], and with the better trends in education reported in adolescents in several countries [18].

We observed no increased risk of maternal adverse outcomes among adolescents compared to adults after adjusting for parity, which showed to be an important confounder. Moreover, we observed a lower risk of hypertensive disorders and obstructed labor among the adolescent groups. The association with lower risk of pre-eclampsia has been previously reported in other studies, including a large hospital-based WHO multi-country study conducted in similar regions [6-8]. The observed lower risk of obstructed labor might be explained by a lower mean birth weight and rates of macrosomia at birth among adolescents [6,7]. Nonetheless, these findings on maternal morbidity outcomes should be interpreted cautiously, due to the limitations described.

The observed increased risk in perinatal outcomes among adolescents compared to adults, with the magnitude of the risk increased in early adolescents is consistent with previous reports. [4-10] However, in our study this finding was not homogeneous across the regions. The neonatal and perinatal mortality in SA was not increased among older adolescents, and the magnitude of the increased risk in preterm birth and LBW was smaller than in SSA and LA. A possible explanation is that the adolescents 15-19 years were actually one year older in SA (mean 18.7 years) than in SSA and LA (mean 17.7 years). However this difference should be considered cautiously, as misclassification of age may be an alternative explanation. In India and Pakistan marriage is not permitted before 18 years of age and it has been reported that families may misrepresent girls as older to avoid this limitation [10]. Nevertheless, it is also plausible that older adolescents in south Asia were qualitatively different than those in SSA and LA. The rates of adolescent pregnancy have been decreasing and are currently low in south Asia, and rarely occur in single women. A possible hypothesis to be tested in other studies would be that this group was more comparable to young adults in their behaviors, and consequently show more comparable outcomes. However this study cannot provide evidence to either support or refute this hypothesis.

The increased risk in perinatal outcomes does not seem to be mediated by a different access of adolescents to antenatal care or a different quality in the care. The differences in access to care are relatively minor among the groups, and the quality measured by the prevalence of screening tests and preventive interventions seems to have been comparable overall to adults. Similarly, the worst perinatal outcomes seem not to be explained by a different access to delivery care; adolescents, and more particularly the younger ones, were more frequently attended by skilled birth attendants and at hospitals than their adult counterparts. We did not have complete enough Registry data to evaluate the nutrition among adolescents and adults in order to explore the possible mediating role of nutritional factors on adverse perinatal outcomes. Consistent with other studies [4,5], our findings suggest that the risks among adolescents cannot be explained only by socio-economic factors, inadequate antenatal or delivery care. Although due to the study nature we cannot rule out residual confounding as a possible explanation of our findings, other unknown age-related factors seem to contribute to the increase perinatal risk in adolescents.

## Conclusions

In summary, this study provides more evidence that pregnancy among adolescents is not associated with worse maternal outcomes, but that it is associated with worse perinatal outcomes, particularly among younger adolescents. However, these trends may differ in regions like south Asia where there are decreasing rates of early adolescent pregnancies. Finally, our study suggests that the increased perinatal risks among adolescents might be associated to other unknown age-related factors, rather than only to socio-economic factors, inadequate antenatal or delivery care.

## Conflicts of interest

The authors declare no conflicts of interest.

## Authors contributions

FA developed the concept and wrote the initial manuscript with input from EMM; JLM performed the analyses, LG, MB, SSG, EC, RJD, AP, SS, OP, FE, and AG oversaw data collection and monitored the study with input from FA, JLM, EAL, KMH, NFK, PLH, RLG, MKT, WAC, MLC, PB, and EMM. JLM performed the statistical analyses with FA and EMM. All authors reviewed and approved the manuscript.

## Peer review

Reviewer reports for this article can be found in Additional file 1.

## Supplementary Material

Additional file 1 Click here for file

## Declarations

This article has been published as part of *Reproductive Health* Volume 12 Supplement 2, 2015: Research reports from the NICHD Global Network for Women’s and Children’s Health Research Maternal and Newborn Health Registry. The full contents of the supplement are available online at http://www.reproductive-health-journal.com/supplements/12/S2. Publication of this supplement was supported by grants from the Eunice Kennedy Shriver National Institute of Child Health and Human Development to RTI International.
