# Supplementary material for: Adverse maternal and perinatal outcomes in adolescent pregnancies: The Global Network’s Maternal Newborn Health Registry study
Source: Reprod Health. 2015 Jun 8;12(Suppl 2):S8. doi: 10.1186/1742-4755-12-S2-S8 (PMC4464033; doi:10.1186/1742-4755-12-S2-S8)
Supplement: Additional file 1 [file 1742-4755-12-S2-S8-S1.pdf]

Referee's comments to the authors– this sheet WILL be seen by the author(s) and published with the article

Title	Adverse maternal and perinatal outcomes in adolescent pregnancies: The Global Network's Maternal Newborn Health Registry study
Author(s)	Fernando Althabe, Janet Moore, Luz Gibbons, Mabel Berrueta, Shivaprasad S Goudar, Elwyn Chomba, Richard Derman, Archana Patel, Sarah Saleem, Omrana Pasha, Fabian Esamai, Ana Garces, Elwyn Chomba, Edward A Liechty, K Michael Hambidge, Nancy F Krebs, Patricia L Hibberd, Robert L Goldenberg, Marion Koso-Thomas, Waldemar A Carlo, Maria L Cafferata, Pierre Buekens, and Elizabeth M McClure on behalf of the Global Network investigators
Referee's name	Tsungai Chipato

When assessing the work, please consider the following points, where applicable:

1. Is the question posed by the authors new and well defined?
2. Are the methods appropriate and well described, and are sufficient details provided to replicate the work?
3. Are the data sound and well controlled?
4. Does the manuscript adhere to the relevant standards for reporting and data deposition?
5. Are the discussion and conclusions well balanced and adequately supported by the data?
6. Do the title and abstract accurately convey what has been found?
7. Is the writing acceptable?

Please make your report as constructive and detailed as possible in your comments so that authors have the opportunity to overcome any serious deficiencies that you find and please also divide your comments into the following categories:

- Major Compulsory Revisions (which the author must respond to before a decision on publication can be reached)
- Minor Essential Revisions (such as missing labels on figures, or the wrong use of a term, which the author can be trusted to correct)
- Discretionary Revisions (which are recommendations for improvement but which the author can choose to ignore)

Where possible please supply references to substantiate your comments.

When referring to the manuscript please provide specific page and paragraph citations where appropriate.

General comments: This manuscript addresses a very topical and important area in obstetrics in a background of numerous previous publications with disparate findings. The strength of this large study is its community level data collection in contrast to many hospital based studies which tend to be biased because the most complicated adolescent pregnancies are managed at referral level.

The study is well designed and the data succinctly described.

The conclusion reached by the authors that the observed increase in perinatal adverse outcomes seems more likely due to biological immaturity does not appear to be supported by any data reported in this study. While biological immaturity might be a plausible cause for preterm delivery, it is more difficult to explain LBW or neonatal death as being related to maternal maturity. The authors might need to expand on how this conclusion was reached.

(continue on the next sheet)

Continued:

Discretional Revisions:

1) It is not clear how the study sites were selected in each country and whether the clusters can be considered as representative of each country in terms of access to education, health care or prevalence of adolescent pregnancy.

2) Nine clusters are listed in the methods section but these appear to have been condensed into seven sites without explanation.

3) The Hierarchical Selection of Confounders is well explained with the aid of Fig 1 but the factors used in analysis are not clear, neither is the selection of two factors-cluster and parity- for adjustment explained.

4) In two rows in Table 1-'Birth attendant' row and 'Delivery location' it is not clear what the figures represent.

Referee's comments to the authors– this sheet WILL be seen by the author(s) and published with the article

Title	Adverse maternal and perinatal outcomes in adolescent pregnancies: The Global Network's Maternal Newborn Health Registry study
Author(s)	Fernando Althabe' Janet Moore, Luz Gibbons, Mabel Berrueta, Shivaprasad S Goudar, Elwyn Chomba, Richard Derman, Archana Patel, Sarah Saleem, Omrana Pasha, Fabian Esamai, Ana Garces, Elwyn Chomba, Edward A Liechty, K Michael Hambidge, Nancy F Krebs, Patricia L Hibberd, Robert L Goldenberg, Marion Koso-Thomas, Waldemar A Carlo, Maria L Cafferata' Pierre Buekens, and Elizabeth M McClure on behalf of the Global Network investigators
Referee's name	UnJa Hayes

When assessing the work, please consider the following points, where applicable:

1. Is the question posed by the authors new and well defined?
2. Are the methods appropriate and well described, and are sufficient details provided to replicate the work?
3. Are the data sound and well controlled?
4. Does the manuscript adhere to the relevant standards for reporting and data deposition?
5. Are the discussion and conclusions well balanced and adequately supported by the data?
6. Do the title and abstract accurately convey what has been found?
7. Is the writing acceptable?

Please make your report as constructive and detailed as possible in your comments so that authors have the opportunity to overcome any serious deficiencies that you find and please also divide your comments into the following categories:

- Major Compulsory Revisions (which the author must respond to before a decision on publication can be reached)
- Minor Essential Revisions (such as missing labels on figures, or the wrong use of a term, which the author can be trusted to correct)

General comments:
This study reconfirms what we know about neonatal outcomes: pregnancy during adolescence leads to poor neonatal outcomes. This article challenges our understanding of pregnancy during adolescence with the unexpected finding of no increased risk of adverse maternal outcomes for younger mothers.

Major compulsory revisions:

- Please provide more justification for the grouping of SSA with LA. The women and context are so different, especially with the inclusion of Argentina (a middle-income country). If group was necessary, I would have expected Guatemala to be grouped with SA due to the size of the women (lower BMI) and likelihood of infection (the absence/lower rate of malaria and HIV).
- It would be helpful to understand how the authors verified the age of the girls/women in the study. Due to existing laws setting limits on age of marriage, it is not unusual for girls, families, and local officials to record girls as older at the time of marriage (and, consequently, delivery).
- Please define adolescence. Definitions can vary greatly, extending as far as the late 20's. Relatedly, it would be helpful to better understand the rationale behind the grouping of the ages. Do the ages of 15 and 20 years mark a specific biological milestone? This may be a source of "noise" in the data. Timing of biological events can vary so much, possibly explaining why there were no differences (at times, only trends) reported for certain outcomes. For example, menarche can range from before 12 years to after 16 years.
- How did the challenges around reliably determining extent of pregnancy influence the study, particularly with the <15 cohort? Were adjustments needed for the younger mothers?
- Was the nutritional status of the girls/women in the three groups different? Could a greater incidence in anemia, infection, and/or low BMI/stunting (undernutrition) explain the increased risk for LBW and preterm delivery in the younger mothers?
- If the differences in the adolescent groups reflect biological immaturity, how does this explain the differences in peri/neonatal mortality between SSA/LA vs. SA adolescents? Biologically, how is 18 different from 19 years old?

Minor essential revisions:

Discretionary revisions:
